# Comparative of *in-vitro* Evaluation between Erlotinib Loaded Nanostructured Lipid Carriers and Liposomes against A549 Lung Cancer Cell Line

**DOI:** 10.22037/ijpr.2019.1100775

**Published:** 2019

**Authors:** Fereydoon Abedi Gaballu, Soheil Abbaspour-Ravasjani, Behzad Mansoori, Reza Yekta, Hamed Hamishehkar, Ali Mohammadi, Gholamreza Dehghan, Behrooz Shokouhi, Shaho Ghahremani Dehbokri, Behzad Baradaran

**Affiliations:** a *Immounology Research Center, Tabriz University of Medical Sciences, Tabriz, Iran.*; b *Student Research Committee, Tabriz University of Medical Sciences, Tabriz, Iran.*; c *Department of Biology, Faculty of Natural Sciences, University of Tabriz, Tabriz, Iran.*; d *Drug Applied Research Center, Tabriz University of Medical Sciences, Tabriz, Iran.*; e *Department of Pathology, Tabriz University of Medical Sciences, Tabriz, Iran.*

**Keywords:** Erlotinib, Liposome, NLCs, A549 cells

## Abstract

Erlotinib (ELT) as a small molecule with poor solubility, poor bioavailability, and instability in gastrointestinal environment, has been considered as a therapeutic agent for Non-Small-Cell Lung Cancer (NSCLC) therapy through oral administration. In the present study, ELT-liposome and ELT-NLCs were successfully prepared and characterized by assessment of the particle size, zeta potential (ZP), polydispersity index (PDI), encapsulation efficiency (EE), and drug loading (DL). DAPI staining and Flow cytometry techniques were employed to probe anticancer activities of the optimal formulations. The obtained results indicated that the average size of optimized ELT-NLCs was 109 ± 2 nm, while the optimal formulation of ELT-liposome was 130 ± 4 nm. In addition, the values of EE, DL, and cellular uptake were higher in ELT-NLCs than ELT-liposome. Moreover, the stability of ELT-NLCs and ELT-liposome were not significantly changed (*P* > 0.05) within storage time. The results of anti-cancer assessment indicated that ELT-NLCs caused more cell viability reduction than ELT-liposome and free ELT. According to the Flow cytometry and DAPI staining results, the exposed A549 cells with ELT-NLCs had more rates of apoptosis than ELT-liposome. The obtained data from this study clearly showed that ELT-NLCs had better anti-cancer activity than ELT-liposome, which may be related to the effective nano particle size, PDI, EE, and DL of ELT-NLCs.

## Introduction

Non-Small Cell Lung Cancer (NSCLC) is the leading cause of death among men and women around the world. NSCLC is probably related to the diverse types of genes mutations such as proto-oncogene B-Raf, anaplastic lymphoma kinase (ALK), discoidin domain receptor tyrosine k 2 (DDR2) and epidermal growth factor receptor (EGFR) (1, 2). However, the overexpression of EGFR protein occurs in NSCLC cells, and activation of it could thoroughly impress tumor cell cycle progression, angiogenesis, invasion, metastasis, and apoptosis. Tyrosine Kinase Inhibitors (TKI) have been represented as a promise method for treatment of NSCLC that occurs via reversible and selective inhibition of EGFR expression (3, 4). Systemic chemotherapy and TKI therapy are initial treatments for rising the survival rate and quality life of the patients (5). In recent years, a small molecule that is named erlotinib (ELT) has been developed and widely investigated as a potent and selective inhibitor for TKI to suppress the expression of EGFR (6). This molecule could bind to the binding site of adenosine triphosphate (ATP) to the tyrosine kinase domain of epidermal growth factor receptor reversibly, and inhibits the auto-phosphorylation process (7). ELT is accessible in oral form for the treatment purposes. The oral pathway is popular method for drugs administration of systemic therapy, but since ELT includes some restrictions such as poor bioavailability (8), instability in the gastrointestinal environment, poor solubility, fatal toxicities (skin rash, Stevens-Johnson syndrome, diarrhoea, etc), and the limited oral administration (9-12).

Therefore, to overcome the mentioned shortages, the development of a new formulation of ELT is essential for clinical domain. In addition, of this, several nanoscale Drug Delivery Systems (DDSs) such as micelle, poly (D, L-lactic-co-glycolic acid(13), liposome (3), dendrimer (14), and mesoporous silica nanoparticles (15) have been recently developed and examined for ELT delivery in the cancer treatment. 

Among the described drug nanocarriers, lipid nanoparticles (LNPs) such as Liposomes, phytosomes, nanostructured lipid carriers (NLCs), etc are available for DDSs of ELT delivery. LNPs as an effective systematic drug administration method is able to enhance the solubilization and stabilization of several drugs by protection of them from the biological fluids, and limitation of nonspecific cytotoxicity (16, 17). It is reported that LNPs contain different advantages that make them a unique oral drug delivery system, including low cost raw materials, exclusively compared with phospholipids, easy scale-up and manufacture, high stability and storage, controlled release, and high versatility (17, 18). Liposome and NLCs are interesting nanocarriers based on LNPs that could deliver their cargoes via encapsulation route (19, 20). The first aim of the present study was to characterize and optimize different formulations of NLC and liposome ELT-loaded systems. The second aim was to study in-vitro anti-cancer activity of the optimized formulations of ELT-liposome and ELT-NLC by assessment of their cellular cytotoxicity, apoptosis, and DNA fragmentations. 

## Experimental

*Materials*

Erlotinib-HCl (ELT) was purchased from Arasto Pharmaceutical Chemicals Inc (Tehran, IRI) miglyol were obtained from Gattefosse (St-Priest, France). Soybean phosphatidylcholine (PC) was achieved from Lipoid GmbH (Ludwigshafen, Germany). Dimethyl sulfoxide (DMSO, 99.9%),3-(4,5-dimethylthiazol-2-yl)-2,5-diphenyltetrazolium bromide (MTT), 4’,6-diamidino-2-phenylindole (DAPI), fetal bovine serum (FBS), RPMI-1640 medium (R5886), phosphate buffered saline (PBS 1X), trypsin (0.25% EDTA solution), poloxamer 407, tween 80Ò, cholesterol and ethanol 99% were supplied from Sigma Aldrich Co. (St. Louis, MO). The annexin V/PI apoptosis detection kit was purchased from Exbio Co. (Exbio, Czech Republic). 

*NLC preparation*

NLC dispersions occurred by the hot homogenization technique. Firstly, ELT was dissolved in 200 m L DMSO. Then, the mixture was added to the melted solid and liquid lipid (Precirol and miglyol, respectively), and then homogenized under 10K rpm speed for 1 minute. Following that, the aqueous surfactant solution (poloxamer 407 with same temperature as melted lipids mixture) was gradually added to the lipid phase less than 23K rpm homogenization (Silent crusher M, Heidolph, Nuremberg, Germany) for 20 min. Then, the hot nano emollition was left in a stagnant place to chill (21).

*Liposome preparation*

A thin layer film was used to produce liposome through hydration-sonication method. Soybean lecithin and cholesterol ratios (90:10, 80:20, 70:30, 60:40, 50:50 and 40:60 w/w) were mixed in 10 mL absolute ethanol as a solvent. Then, 5 mg of ELT was added to the lipid solution and mixed until complete dissolution. Then, the lipid/drug solution was poured into the round balloon. To provide the thin layer, the solvent was removed from the mixture by a rotary evaporator at 40 ^o^C. The obtained film was hydrated by 10 mL of 1X PBS solution at 65 ^o^C for 8 min. To reduce the size of the provided film, the formulation was exposed to a probing sonication (Vibra Cell-Sonics & Material, 130 W, 20 kHz, USA) at 80% sonication strength under ice bath for 10 min (10 cycles of 1 min sonication and 1 min rest for allowing the samples to cool down) (22).

*Size and zeta potential (ZP) analyses*

The size and ZP of the nano liposomes and NLCs were measured by a dynamic light scattering (DLS) method using Malvern zeta sizer (Malvern Instruments Ltd., Malvern, UK) at the room temperature. All samples were diluted 10 times with purified water. All measurements were done in triplicate.

*Scanning electron microscope (SEM)*

The shapes and morphology of the prepared formulations were monitored by scanning electron microscope (TEscan, VEGA II XMU, Czech Republic). Before scanning, the samples were coated with gold, using a direct current sputter technique (DST1, Nanostructured coating co, Iran).

*Encapsulation efficiency and drug loading*

The EE parameter was determined by measurement of the concentration unentrapped ELT in NLCs and liposomes. The Amicon^®^ Ultra-4 100 k – a 30 kDa molecular weight cut-off membrane (Millipore, Billerica, MA) was employed to this purpose. Briefly, 2 mL of each samples (ELT liposome and NLCs) were placed into the Amicon filter and centrifuged under 4K rpm for 10 min. The concentration of the unentrapped (unloaded) drug was determined by a validated UV–vis spectrophotometric method (Ultrospec 2000 UV/Visible, Parmacia Biotech Instruments Ltd, Cambridge, England) at 343 nm. The rate of DL and EE was calculated using the following equations (Equations 1 and 2), respectively:

$\mathrm{EE}(\%)=\frac{C_{T}-C_{\mathrm{AP}}}{C_{T}}\times 100$

Equ. 1

$\mathrm{DL}(\%)=\frac{W_{\mathrm{DL}}}{W_{\mathrm{NP}}}\times 100$

Equ. 2

where C_T_, C_AP_, W_DL_, and W_NP_ represent total concentration of the added drug, concentration of drug in the aqueous phase, weight of the loaded drug into NPs, and solid mass weight of NPs, respectively (23).

*Stability study*

Stability of ELT-liposome, ELT-NLCs was done by measuring changes in the particle size, ZP, and PDI. All of the samples were stored at refrigerator at 4-8 C^o^ temperature for 30 days. 

*In-vitro cellular uptake*

Rhodamine B (RhB) as a fluorescent agent moiety was employed to confirm cellular uptake of the prepared formulations by A549 cells. To this purpose, RhB was added to the NLC formulation (0.5% w/w rhodamine B ratio to lipid) in the melted lipid phase stage. After a complete mixing, the aqueous phase was added to it. The separation fluorescent of NLC from free florescent was done by using the Amicon tube (Ultra-30 kDa molecular weight cut-off membrane, Millipore, Germany). For this aim, the fluorescent NLCs were placed into the upper chamber of Amicon filter and centrifuged under 4k rpm for 15 min. This process was performed to ensure thoroughly elimination of un-entrapped rhodamine B from NLC formulation (24). In the fluorescent - liposome purification, RdB was add to the liposome in construction of thin layer film step and encapsulated by liposome to mimic the encapsulation of the ELT. Next process of this purification has been done similar to the NLCs formulations (25). A549 cells (5 x 10^4^ per well) were seeded in six-well, after that the cells were treated with 1, 10, 20, 40, 80, and 160 µg/mL of RhB labeled NLCs and liposome formulations for 4h, when the cells reached to 70% confluency. The quantitative cellular uptake of the prepared formulations was measured by a flow cytometry. 

*Cytotoxicity study*

MTT (3-[4, 5-dimethyl-2-thiazolyl] - 2, 5 diphenyl tetrazolium bromide) assay was carried out to probe the effects of free ELT, ELT-NLCs, and ELT-liposome formulations on the viability of A549 cells (26). Briefly, 15×10^3^ three time passaged A549 cells were seeded into each well of 96-well plate. Then, the cells were treated with different concentrations of free ELT (10-60 µg/mL) and its nano formulations. After 24 and 48 h, 50 mL of MTT solution (2mg/mL PBS) was added to each well. Incubated medium was replaced with 200 m L of DMSO after 4 h. Then, the absorption of the plates was read at 570nm by a TCAN Elisa Reader.

*DAPI staining*

DAPI staining is a method that represents the chromatin fragmentation. To implement this assay, A549 cells were seeded into six-well plates, including 12mm cover-slips at seeding density of 5 X 10^5^ cells per well. The cultivated cells were treated with free ELT, blank NLCs and liposomes, ELT-liposome, ELT-NLCs, and docetaxel (300 µg/mL) as a positive control for 48 h. Then, the cells were fixed with 4% formaldehyde for 3 h. Following that, the cells were permeabilized with 0.1% Triton X-100 in PBS for 5 min. Next, the cells were stained with DAPI 0.1% for 10 Min. Finally, the studied cells were monitored by fluorescence imaging system (Cytation 5, Biotek, USA). 

*Flow cytometry*

To measure the rate of cell apoptosis, A549 cells were seeded into six-well plate at a density of 5 × 10^5 ^cells per well in the RPMI 1640 media with 10% FBS. After 24 h, the media was changed with 2 mL fresh media, contained IC_50_ concentration of each treatment., and the cells were incubated for 48 h. 

Following that, the cells were detached and stained via annexin V/PI apoptosis detection kit (Exbio, Czech Republic) according to the manufacture’s protocol. Then, the rate of apoptosis was analyzed by flow cytometer equipment (MACS Quant 10, Miltenyi Biotech GmbH). The achieved data was analyzed by using FlowJo software package (Treestar, Inc., San Carlos, CA) (27). 

*Statistical analysis*

All parameters were expressed as mean ± standard deviation. Statistical analyses were carried out using one-way and two-way analyses of variance (one-way and two-way ANOVA) with multiple comparisons between deposition data using a LSD significant difference test (Prism, version 7.0, Graphpad softwhere, INC). A *P *_value_ less than 0.05 was considered as significant.

## Results

*ELT loaded liposome and NLC characterization*

**Figure 1:**
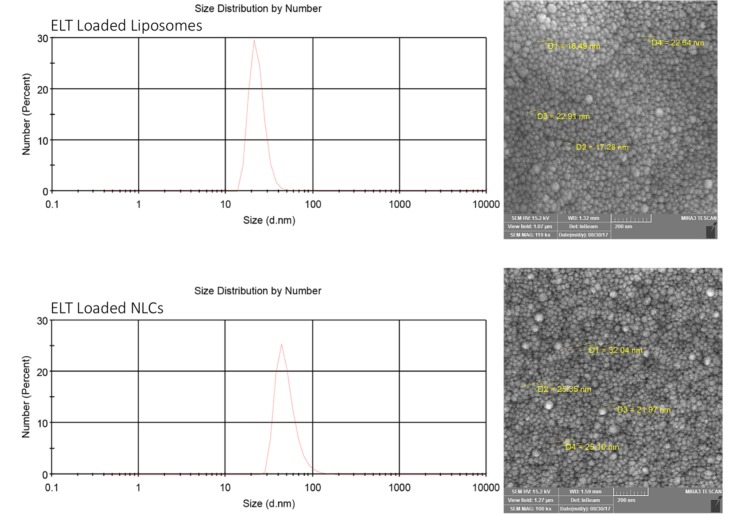
Represents size analysis and SEM imaging of ELT loaded Liposomes (a) and ELT loaded NLCs (b)

According to Table 1, the optimal ELT – NLCs formulation (F_4_) was made from 80 mg of precirol, 20 mg of miglyol and 3% poloxamer (the size of 110 nm, -16 zeta potential (ZP), 0.22 PDI, 87% EE and 4.35% DL). Furthermore, the optimal ELT-liposome formulation (Table 2, F_1_) was provided from 90 mg of phosphatidylcholine, 10 mg of cholesterol and 10 mg of tween 80. The optimal ELT- liposome formulation was 130 ± 4 nm, with an -18 ZP and 0.54 PDI. Furthermore, EE and DL factors of ELT- liposome formulation were calculated to be approximately 68% and 3.4%, respectively (Table 2). The particle size measured by SEM for the optimal ELT-NLCs and ELT- liposome formulations were 22 to 100 nm and 36 to 110 nm, respectively (Figure 1).

**Table 1:** Composition, particle size, zeta potential, polydispersity index (PDI), encapsulation efficiency (EE), drug loading (DL) and formulation method of ELT-loaded NLCs. The results were calculated as the mean ± standard deviation (n = 3) *Precirol: Miglyol

**Formulation code**	**Method**	**PC:MG** **(W/W)** *	**Ploxamer**	**Drug (mg)**	**Size (nm)**	**Zeta**	**PDI**	**EE (%)**	**DL (%)**
F1	HH	90:10	1%	5	928±17	-16±8	1.21±0.41	49±4.73	2.45±0.21
F2	HH	80:20	1%	5	761±11	-10±4	0.81±0.21	52±5.25	2.60±0.26
F3	HH	90:10	3%	5	144±5	-11±2	0.35±0.21	79±2.15	3.95±0.10
F4	HH	80:20	3%	5	109±2	-16±5	0.22±0.11	87±3.11	4.35±0.15
F5	HH	90:10	5%	5	238±9	-13±3	0.41±0.09	59±2.21	3.45±0.11
F6	HH	80:20	5%	5	221±16	-19±7	0.99±0.34	61±1.81	2.05±0.09

**Table 2:** Composition, particle size, zeta potential, polydispersity index (PDI), encapsulation efficiency (EE), drug loading (DL) and formulation method of ELT-loaded liposomes. The results were calculated as the mean ± standard deviation (n = 3). *Phosphatidylcholine: Cholesterol: Twin 80Ò

**Formulation code**	**Method**	**PC:Ch:T** *	**Drug (mg)**	**Size (nm)**	**Zeta**	**PDI**	**EE (%)**	**DL (%)**
F1	HH	90:10:10	5	130±4	18±1	0.54±0.09	68±1.14	3.4±0.05
F2	HH	80:20:10	5	148±8	12±3	0.29±0.13	38±2.64	1.9±0.13
F3	HH	70:30:10	5	192±6	19±4	0.41±0.23	41±3.14	2.05±0.15
F4	HH	60:40:10	5	291±10	9±2	0.51±0.12	31±6.1	1.55±0.30
F5	HH	50:50:10	5	581±18	4±3	0.41±0.10	48±2.62	2.4±0.13
F6	HH	40:60:10	5	748±21	8±6	0.47±0.13	41±3.51	2.05±0.17

**Table 3:** The IC50 concentration of free ELT, ELT loaded Liposomes and NLCs in 24 and 48 h. The results were calculated as the mean± standard deviation (n = 3). (*P < 0.05, **P < 0.01).

**Times**	**Free Drug**	**ELT loaded liposome**	**ELT loaded NLC**
24	49.11 ± 3.21	31.87 ± 5.28 *	13.04 ± 4.16 **
48	51.85 ± 4.23	10.07 ± 1.87 **	4.224 ± 0.32 **

**Figure 2:**
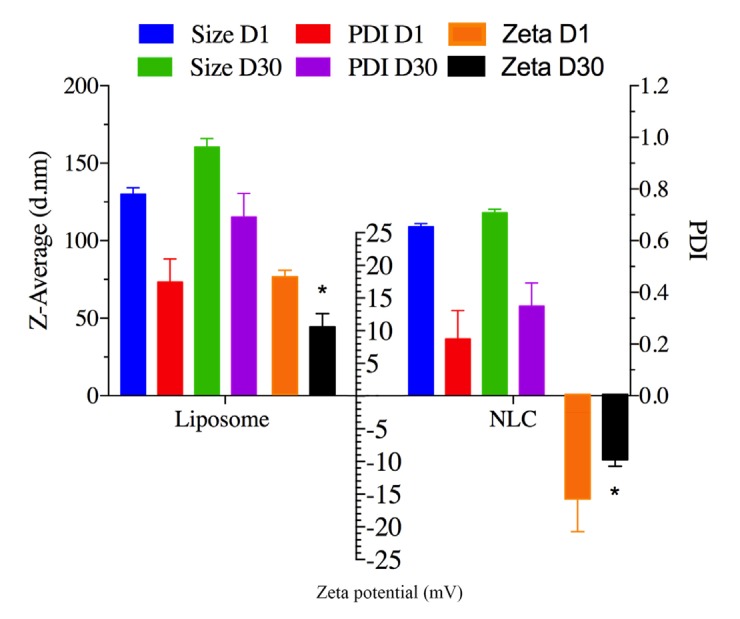
Showing the storage stability of ELT loaded Liposomes and NLCs in refrigerator at 4-8 oC. The results were calculated as the mean ± standard deviation (n = 3). (*P < 0.05, **P < 0.01)

**Figure 3:**
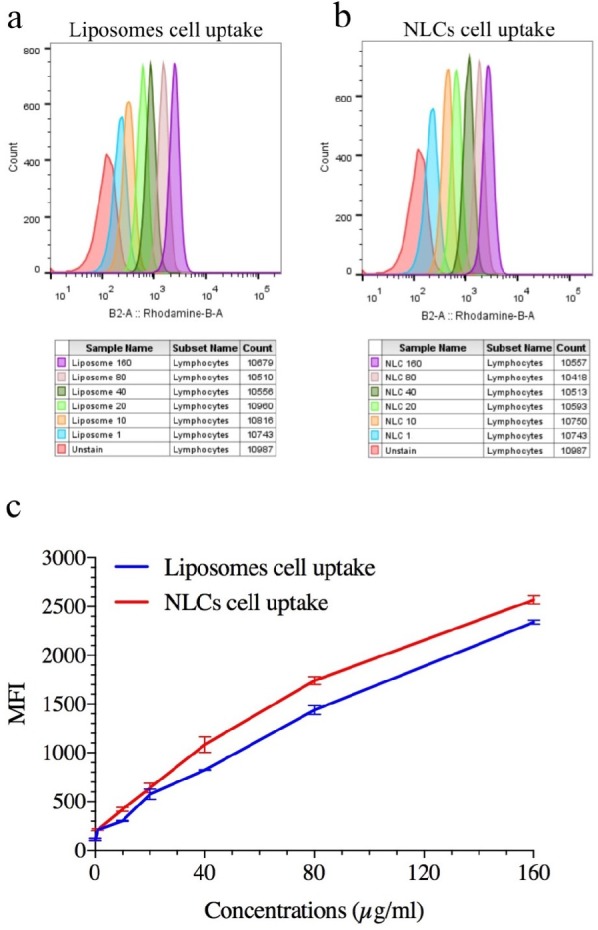
Representation Quantitative cellular uptake of ELT-NLCs and ELT-liposome by A549 cell line that is measured by flow cytometry. A) ELT-liposome formulation cellular uptake. B) ELT-NLCs formulation cellular uptake and C) MFI of ELT-NLCs and ELT- liposome formulations. The results were calculated as the mean ± standard deviation (n = 3)

**Figure 4:**
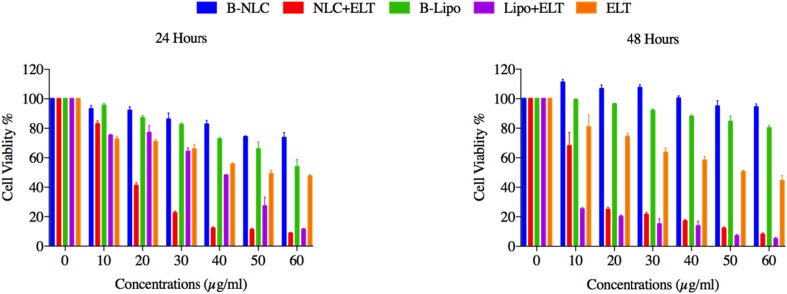
Represents *in-vitro *cellular viability results of free ELT, ELT loaded Liposomes and NLCs in A549 cell line over 24 and 48 h. The results were calculated as the mean ± standard deviation (n = 3).

**Figure 5:**
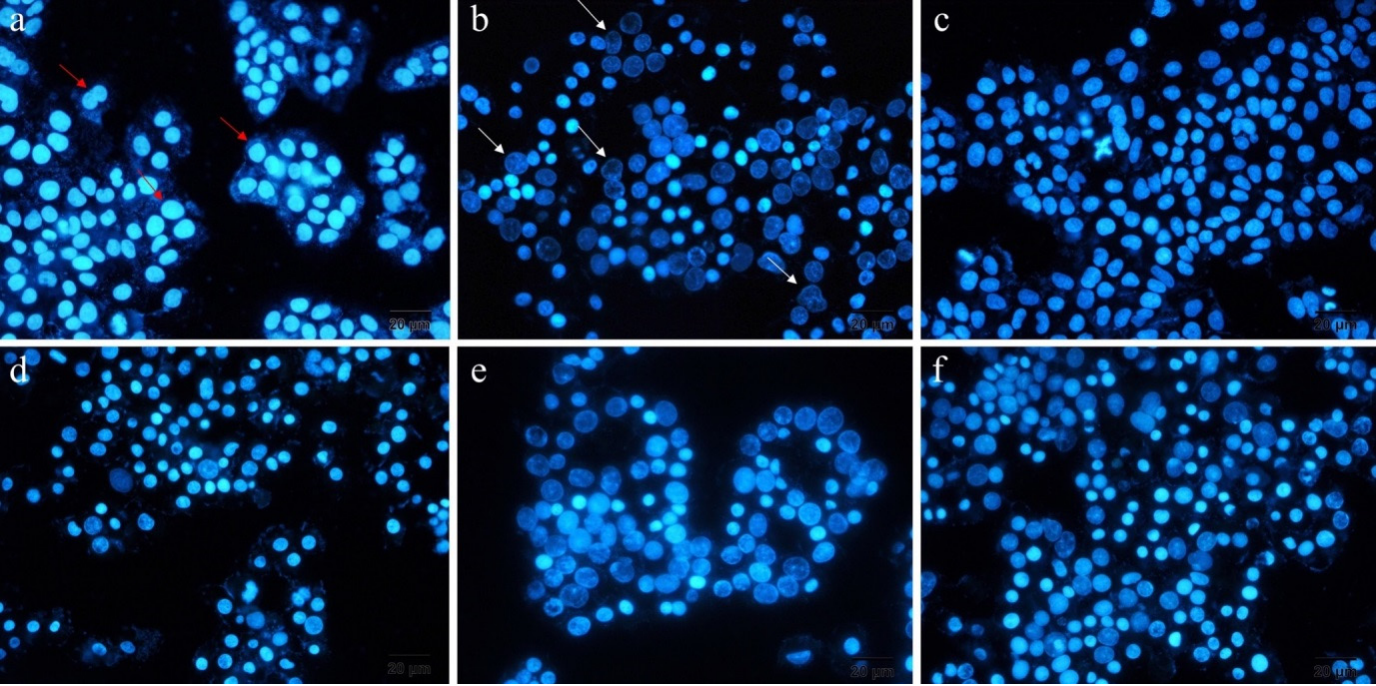
Displays fluorescent images of treated and untreated DAPI stained A549 cells, a) untreated, b) treated with docetaxel, c) blank, d) free drug, e) ELT loaded liposomes and f) ELT loaded NLCs (Red arrows shown healthy cell nuclei and white arrows shown fragmented cell nuclei samples

**Figure 6:**
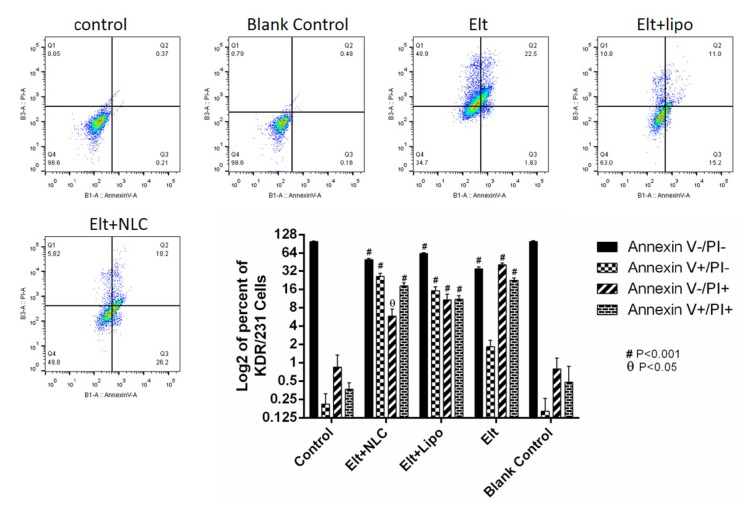
Illustrates cell apoptosis induced by free ELT, ELT loaded Liposomes and NLCs over 48 h by using Annexin V-FITC/PI staining. The results were calculated as the mean ± standard deviation (n = 3). (ØP < 0.05, #P < 0.01)

*Storage stability study*

ELT- NLCs and ELT- liposome formulations were stored at 4-8 C^o^. The particle size and PDI were measured to approve or disapprove the stability of the stored samples on 1^st^ day and 30^th^ day. As it is shown in Figure.2, there was not a significant (*P* > 0.05) difference in the particle size of formulations over the storage period. However, ZP parameters of both optimal formulations showed a significant difference (*P *< 0.05) between 1^st^ and 30^th^ days. 

*In-vitro cellular uptake*

Quantitative cellular uptake was carried out to monitor the cellular internalization of rhodamine B labeled NLCs and liposome formulations. The flow cytometric histogram showed that both NLC and liposome formulations had remarkable cell uptakes, and their internalizations were performed dose-dependent manner (Figures 3 A, B). On the other hand, the comparison of Mean Fluorescent Intensity (MFI) of fluorescent-labeled NLCs and liposome formulations exhibited that NLC nanoparticle had significantly (*P* <0.05) internalization compared with liposome formulation (Figure 3 C).

*In-vitro cell viability and IC50 of nano formulations*

The effects of free ELT, ELT-liposome and ELT- NLCs formulations on the cells viability were measured using MTT assay. A significant (*P* < 0.05 and *P* < 0.01) reduction was distinguished in in the cells viability, after 24 and 48 h of free ELT and nano formulations treatment. It was shown that 50 µg and 20 µg of ELT-liposome were able to decrease the cells viability close to 20% during 24 and 48 h, respectively, while 30 µg and 10 µg of ELT-NLCs decreased the cells viability around %20 over 24 and 48 h, respectively. It was indicated that 60 µg of free ELT was the most effective dosage in the reduction of 50% cell viability over both 24 and 48 h Figure 4. The IC_50_ values of ELT-liposome and ELT-NLCs were 31.87 and 13.04 µg during 24 h, respectively. In addition, The IC_50 _values of ELT-NLCs and ELT-liposome were 4.22 and 10.07 µg over 48 h, respectively. These results indicate that there is a significant difference between the IC_50 _rates of free ELT (49.11 µg) and loaded ELT (Table 3).

*DAPI Staining*

In this study, DAPI staining assay was done for recognition of DNA fragmentation in nucleus of the treated A549 cells with free ELT, ELT-liposome, and ELT-NLCs formulations Figure. 5 From the morphology assessment of DAPI stained cells, free ELT resulted in low deformation of DNA fragmentation, while DNA fragmentation was considerably increased when the cells were treated with ELT- liposome and ELT- NLCs formulations.

*Flow-cytometry*

After treatment of A459 cells with ELT and its nano formulations, they were stained with annexin V/PI to evaluate the rate of apoptosis over 48 h. In this study, the non-treated cells were considered as a negative control group. As it is shown in Figure 6, the treated A549 cells with blank NLPs were negative in this assay. According to the obtained results, free ELT exhibited 41% necrosis and 24% apoptosis, while ELT- liposome formulation showed 11% necrosis and 27% apoptosis. In addition, the treated A549 cells with ELT- NLCs formulation had 6% necrosis and 44% apoptosis.

## Discussion

In the present study, two different ELT loaded delivery systems, including ELT-liposome, ELT-NLC formulations were prepared, and the optimal formulations were selected. The anti-cancer activities of the provided formulations were compared with each other on A549 cancer cell line. The results of this study indicated that ELT-NLCs had appropriate physicochemical characteristic and better anti-cancer activities compared with ELT- liposome. 

Generally, the internalization of nanoparticles into cells is strongly associated with their physicochemical characteristics, shape, and total charges (28). The size of nanoparticle is an important factor, which can affect the pharmacological features of the particles. As listed in Table 1 and 2, the size of both ELT-NLCs and ELT-liposome was affected by their compositions (different rates of lipids). The average size of the optimized formulation of ELT-NLCs was considerably lower than ELT-liposome, that was probably attributed to poloxamer 407 as surfactant on the surface of NLC particles. The main role of surfactant is known to have ability of stabilizing nanoparticles in the colloidal condition as well as prevention of aggregation process over the storage. The selection of stabilizers is a crucial factor for formulation of a nanoparticle due to their important roles in controlling the size and stabilization of the dispersions (21, 29). Therefore, the lower PDI values of ELT-NLC than ELT-liposome are justifiable. Moreover, EE and DL were acceptable for both of the optimized formulations; that is may be associated with strong hydrophobicity of ELT. However, ELT- NLCs formulation showed the higher rates of EE and DL in comparison with ELT- liposome formulation. SEM imaging was carried out to show spherical structure, shape, and smooth of the particles. The spherical shapes of the both optimal formulations were shown in Figure.1, confriming the encapsulation of ELT did not induce morphological changes. Moreover, SEM imaging data represented a relatively uniform size distribution for ELT- NLCs and ELT- liposomes formulations that were approved by DLS. The ZP parameter of ELT- NLCs formulation were negative, while these parameters were represented positive for ELT- liposome formulation. 

Figure 2 represents the particle size, ZP, and PDI of the nano formulations as stability factors. There were slightly changes in the particle size of the formulations over the storage period. However, the ZP parameters of both formulations significantly changed at the storage time. It is suggested that both optimal formulations are stable over the storage time, and stability of the formulations may be due to the presence of steric repulsion of the poloxamer 407 and tween 80 as surfactants in the structures of NLC and liposome structures, respectively. 

Encapsulation of therapeutic agents can reduce their toxicity through various mechanisms such as the increment of drug penetration into targeted cells, modulation of drug release profile, and high endocytosis phenomenon (30). The viability of A549 cells, that were incubated with blanks of liposome and NLC for 24 and 48 h was around 80%, indicating that these blanks have lower cell cytotoxicity. These results revealed that ELT- NLCs and ELT- liposome formulations could significantly (*P* < 0.05) reduce the cells viability rate in comparison with free ELT, that may be attributed in easily internalization of ELT- NLCs and ELT- liposome formulations. The results indicated that ELT- NLCs formulation was significantly able to decrease cell viability over 24 and 48 h compared with ELT- liposome formulation, which could be explained by smaller size of ELT- NLCs. These results are consistent with previous reports (31). 

According to the results the IC_50_ values of A549 cells incubated with ELT- NLCs and ELT- liposome formulations were calculated 3.8 and 1.5 times lower than free ELT during 24 h, respectively. Therefore, it could be concluded that the encapsulation of ELT through liposome and NLC could improve its cellular cytotoxicity. Furthermore, the comparison of the IC_50_ values among free ELT, ELT- NLCs, and ELT- liposome formulations indicated that ELT- NLCs formulation was 2.5 and 12 times lower than ELT- liposome formulation and free ELT over 48 h, respectively. 

From flow cytometry data, the rate of necrosis on the treated A549 cells with free ELT was approximately two times higher than the apoptosis rate. The encapsulation of ELT by liposomes and NLCs could markedly decrease the rate of necrosis. A549 cells treated with blank did not show any apoptosis and necrosis, shown the safety of used blank. On the other hand, ELT-NLCs formulations were able to significantly induce A549 cell apoptosis in comparison with ELT-liposome and free ELT, which could be related to its higher cellular uptake. Quantitative cellular uptake measurement showed an increase dose-dependent cellular internalization of the formulations that could be associated to the enhanced endocytosis archived in the interaction among ELT-NLCs and ELT-liposome formulations with A549 cell membrane. As shown in Figure 3-C, A549 cells demonstrated that also both of ELT-NLCs and ELT-liposome formulations could internalize into the A549 cells with higher intensity but ELT loaded NLCs formulation had strongly cellular uptake. In addition, flow cytometry results were approved by DAPI investigation. Our results demonstrated that free ELT, ELT- NLCs, and ELT- liposome formulations are able to induce apoptosis, which could be detected from the morphological changes of nucleus. ELT- NLCs formulation represents higher rate of apoptosis activation ability than ELT- liposome formulation. These results are consistent with the idea that the progression of apoptosis is linked with the high endocytosis.

## Conclusion

t study, the authors successfully prepared different formulations of ELT-liposome and ELT-NLC. The optimal formulations were selected according to their physiochemical characterizations data. We found that the optimal formulation of ELT-NLCs had markedly suitable physiochemical characteristic than ELT-liposome due to having small size, PDI with a higher EE and DL compared with ELT-liposome. Further, the optimal formulation of ELT-NLCs showed better cellular uptake and anti-cancer activity than ELT-liposome formulation.
